# Plasma proteomic signatures of childhood adversity

**DOI:** 10.1093/procel/pwag028

**Published:** 2026-04-22

**Authors:** Jiening Yu, Duoduo Fu, Xucheng Wu, Weijing Gao, Hongwei Chen, Xueqing Jia, Fan Pu, Yanjie Zhao, Kaili Sun, Liming Zhang, Wen Wang, Huiqian Huang, Anna Dearman, Zuyun Liu

**Affiliations:** Center for Clinical Big Data and Analytics Second Affiliated Hospital, Department of Big Data in Health Science School of Public Health, Zhejiang Key Laboratory of Intelligent Preventive Medicine, Zhejiang University School of Medicine, Hangzhou 310058, China; Department of Epidemiology, Harvard T.H. Chan School of Public Health, Boston, MA 02115, United States; Center for Clinical Big Data and Analytics Second Affiliated Hospital, Department of Big Data in Health Science School of Public Health, Zhejiang Key Laboratory of Intelligent Preventive Medicine, Zhejiang University School of Medicine, Hangzhou 310058, China; Center for Clinical Big Data and Analytics Second Affiliated Hospital, Department of Big Data in Health Science School of Public Health, Zhejiang Key Laboratory of Intelligent Preventive Medicine, Zhejiang University School of Medicine, Hangzhou 310058, China; Center for Clinical Big Data and Analytics Second Affiliated Hospital, Department of Big Data in Health Science School of Public Health, Zhejiang Key Laboratory of Intelligent Preventive Medicine, Zhejiang University School of Medicine, Hangzhou 310058, China; Center for Clinical Big Data and Analytics Second Affiliated Hospital, Department of Big Data in Health Science School of Public Health, Zhejiang Key Laboratory of Intelligent Preventive Medicine, Zhejiang University School of Medicine, Hangzhou 310058, China; Center for Clinical Big Data and Analytics Second Affiliated Hospital, Department of Big Data in Health Science School of Public Health, Zhejiang Key Laboratory of Intelligent Preventive Medicine, Zhejiang University School of Medicine, Hangzhou 310058, China; Center for Clinical Big Data and Analytics Second Affiliated Hospital, Department of Big Data in Health Science School of Public Health, Zhejiang Key Laboratory of Intelligent Preventive Medicine, Zhejiang University School of Medicine, Hangzhou 310058, China; Center for Clinical Big Data and Analytics Second Affiliated Hospital, Department of Big Data in Health Science School of Public Health, Zhejiang Key Laboratory of Intelligent Preventive Medicine, Zhejiang University School of Medicine, Hangzhou 310058, China; Center for Clinical Big Data and Analytics Second Affiliated Hospital, Department of Big Data in Health Science School of Public Health, Zhejiang Key Laboratory of Intelligent Preventive Medicine, Zhejiang University School of Medicine, Hangzhou 310058, China; Department of Health Policy and Management, Yale School of Public Health, New Haven, CT 06520, United States; Institute for Social and Economic Research, University of Essex, Colchester, Essex CO4 3SQ, United Kingdom; Basic and Translational Research Center, The Second Affiliated Hospital, Zhejiang University School of Medicine, Hangzhou 310058, China; Institute for Social and Economic Research, University of Essex, Colchester, Essex CO4 3SQ, United Kingdom; Center for Clinical Big Data and Analytics Second Affiliated Hospital, Department of Big Data in Health Science School of Public Health, Zhejiang Key Laboratory of Intelligent Preventive Medicine, Zhejiang University School of Medicine, Hangzhou 310058, China

**Dear Editor,** 

Childhood adversity, typically referring to experiences of emotional, physical, and sexual abuse and/or emotional and physical neglect, is a critical early-life risk factor with far-reaching implications for health. Childhood adversity has been linked to limited types of diseases (e.g., mental disorders and respiratory disease), as detailed in a systematic review and meta-analysis (Hughes et al., 2017). However, the impacts of childhood adversity on a broader spectrum of diseases (e.g., musculoskeletal and genitourinary diseases) and the underlying biological mechanisms remain inadequately explored, hindering the development of preventive programs and targeted interventions. Emerging sporadic evidence suggests that childhood adversity can influence disease patterns through its impacts on molecular alterations such as DNA methylation (Lussier et al., 2024), telomere length (Yu et al., 2024), and clinical biomarkers (e.g., C-reactive protein) (Alley et al., 2025), providing limited insights into the biological embedding of childhood adversity. The plasma proteome, which reflects the complex interplay of genetic and environmental factors that contribute to pathophysiological processes, has become an essential tool for investigating the mechanisms underlying disease development in recent years (Deng et al., 2025). Therefore, it is essential and meaningful to address the following questions: How does childhood adversity influence plasma proteins? Does it establish a causal relationship? Are there specific proteins that mediate the long-term impacts of childhood adversity on diseases? Could these proteins be leveraged to develop a proteomic score for assessing childhood adversity objectively?

Here, leveraging a broad spectrum of diseases and large-scale plasma proteome data from the UK Biobank (UKB), we primarily aimed to: (i) comprehensively investigate the impacts of childhood adversity on 1,045 diseases (396 prevalent and 649 incident) across 14 disease categories (median follow-up: 13.8 years), (ii) systematically characterize the plasma proteomic signatures of childhood adversity through proteome-wide association study (PWAS), pathway enrichment, protein–protein interaction network (PPI), protein co-expression network, and Mendelian randomization (MR) analyses, (iii) examine whether and to what extent the plasma proteins mediate the associations between childhood adversity and diseases, and (iv) develop a novel proteome-based measure of childhood adversity, termed as proteomic childhood adversity score (PCAS), and evaluate its predictive performance for incident diseases.

In the UKB, 150,174 (analytic sample 1; 56.3% female) and 12,933 (analytic sample 2, a subsample of analytic sample 1; 55.9% female) participants were included in the analyses to characterize the broad disease spectrum and plasma proteomic signatures of childhood adversity (Fig. S1). The mean (standard deviations [SDs]) ages of participants in analytic samples 1 and 2 were 56.4 (7.7) years and 56.5 (7.8) years, respectively (Table S1). The majority were White (97.2% and 96.9%, respectively). Childhood adversity was assessed using the five-item Childhood Trauma Screener, yielding a self-reported childhood adversity score ranging from 0 to 5. In both analytic samples, females had higher childhood adversity scores and a higher prevalence of most childhood adversity subtypes (including physical neglect, emotional neglect, sexual abuse, and emotional abuse), with the exception of physical abuse. Comparisons between the two analytic samples showed modest differences in age, race and ethnicity, occupation, and Townsend deprivation index (TDI), whereas sex, educational level, body mass index (BMI), lifestyle factors, and childhood adversity were comparable (Table S2). The detailed basic characteristics of study participants and the flow chart of analytic samples are presented in Tables S1 and S2 and Fig. S1, respectively.

We investigated the associations between childhood adversity and 396 prevalent diseases using logistic regression models and 649 incident diseases by Cox proportional hazards models in analytic sample 1. Models were adjusted for baseline age, sex, race and ethnicity, educational level, occupation, TDI, BMI, smoking status, drinking status, and exercise status. After false discovery rate (FDR) correction (*P*_FDR_ < 0.05), childhood adversity was significantly associated with 198 prevalent diseases and 338 incident diseases across 14 disease categories, most notably psychiatric disorders. Specifically, for prevalent diseases, childhood adversity was most significantly associated with any mental disorder (odds ratio [OR] = 1.31 per 1-point increase in childhood adversity score; 95% CI: 1.26, 1.35) (Fig. S2; Table S3). For incident diseases, mood disorders (hazard ratio [HR] = 1.31, 95% CI: 1.29, 1.33), depression or dysthymia (HR = 1.31, 95% CI: 1.28, 1.33), depression (HR = 1.30, 95% CI: 1.27, 1.32), any mental disorder (HR = 1.19, 95% CI: 1.18, 1.21) and anxiety disorders (HR = 1.26, 95% CI: 1.24, 1.29) demonstrated the top significant associations with childhood adversity (Fig. S2; Table S4). Additionally, we observed that childhood adversity was significantly associated with musculoskeletal, gastrointestinal, respiratory and genitourinary diseases, particularly among prevalent diseases (Fig. S2; Table S3).

Considering possible sex-specific effects of childhood adversity, sex-stratified analyses were conducted. A total of 94 prevalent diseases and 152 incident diseases were significantly associated with childhood adversity in males, and 171 prevalent diseases and 306 incident diseases in females (*P*_FDR_ < 0.05). Consistent with the primary findings, psychiatric disorders exhibited the strongest associations with childhood adversity in both sexes, with more pronounced associations in females (Fig. S3; Tables S5–8). In sensitivity analyses, 62.1% (123/198) of prevalent diseases and 77.2% (261/338) of incident diseases remained significantly associated with childhood adversity (*P*_FDR_ < 0.05) after additional adjustment for comorbidity status at each disease endpoint (Fig. S3; Tables S9 and S10).

Through PWAS, we investigated the plasma proteomic signatures of childhood adversity in analytic sample 2, adjusting for the same covariates as in the above analyses. Out of 2,919 proteins, 381 proteins were significantly associated with childhood adversity (*P *< 0.05), of which 39 remained significant after FDR correction (Fig. 1A; Table S11). Most associations were positive, indicating that participants experiencing greater childhood adversities exhibited higher protein abundance. Polymeric immunoglobulin receptor (PIGR), which plays a key role in microbial homeostasis and mucosal immunity, exhibited the most significant association with childhood adversity (β  =  0.017 per 1-point increase in childhood adversity score; standard error [SE] = 0.003), followed by gastrin (GAST), a critical protein participating in all major gastrointestinal activities including secretion, motility and absorption, which exhibited the second most significant association with childhood adversity (β  =  0.046, SE = 0.009).

**Figure 1.**
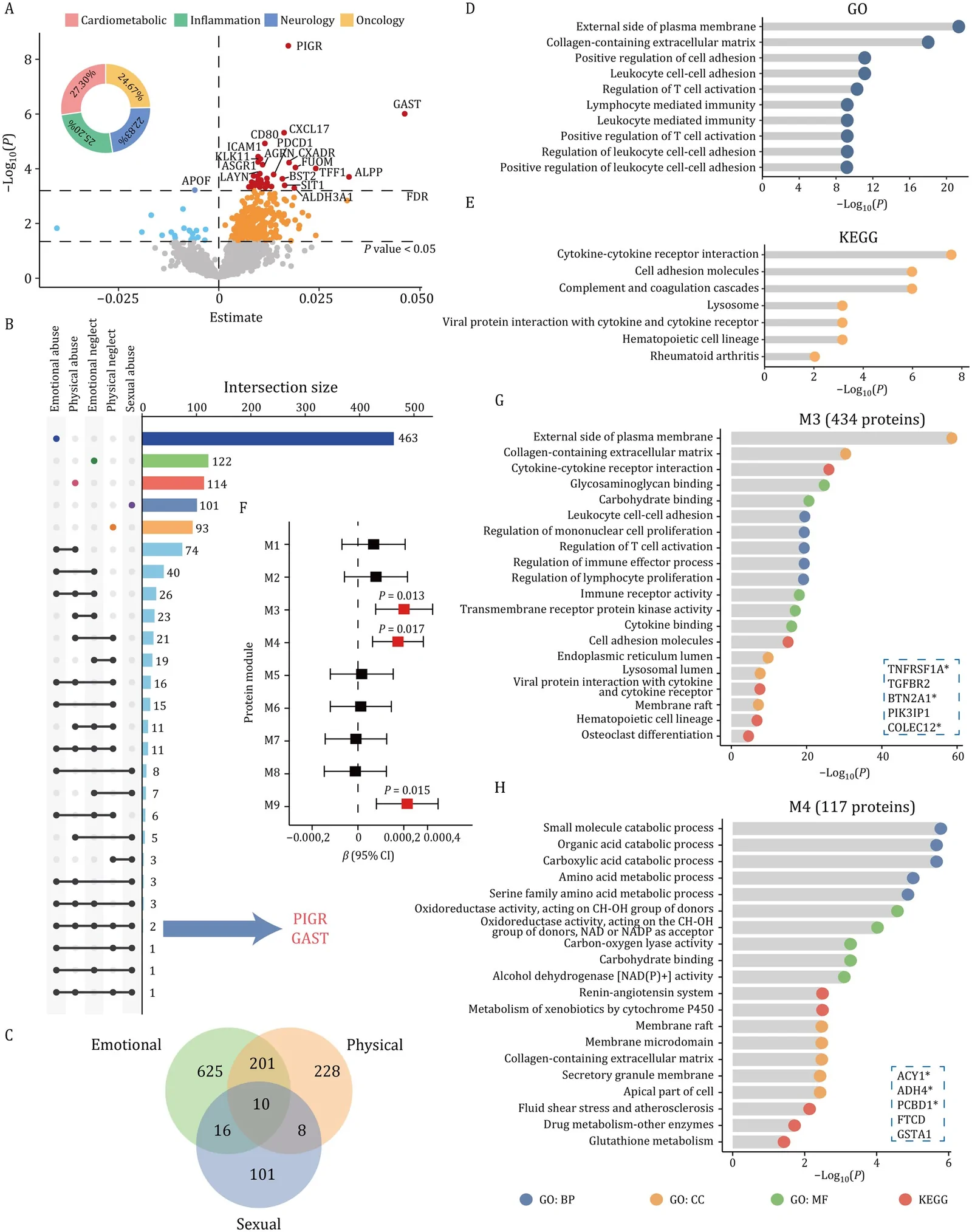
**Plasma proteomic signatures of childhood adversity**. (A) Associations of childhood adversity with protein levels. Linear regression models were performed, adjusted for age, sex, race and ethnicity, educational level, occupation, Townsend deprivation index, body mass index, smoking status, drinking status and exercise status. The x-axis represents βs, and the y axis represents −log_10_[*P* values]. The pie charts depict the proportions of 381 identified proteins across four distinct panels. (B) UpSet diagram showing the number of proteins associated with five subtypes of childhood adversity (i.e., physical neglect, emotional neglect, sexual abuse, physical abuse, and emotional abuse) and the overlaps among these associations. PIGR and GAST were simultaneously associated with five subtypes. (C) Venn diagram showing the number of proteins associated with three subtypes of childhood adversity (i.e., emotional abuse and neglect, physical abuse and neglect, and sexual abuse) and the overlaps among these associations. (D) The top 10 enriched GO CC, BP and MF for proteins linked to childhood adversity. (E) The top seven enriched KEGG pathways for proteins linked to childhood adversity. The x-axis represents −log_10_[*P* values]. (F) Associations between childhood adversity and nine protein modules (refer to M1–M9). Linear regression models were performed, adjusted for age, sex, race and ethnicity, educational level, occupation, Townsend deprivation index, body mass index, smoking status, drinking status, and exercise status. The x-axis represents β coefficients and the error bars indicate 95% CIs. The red markers demonstrate significance after Bonferroni correction (*P *< 0.05/9 = 0.006). (G and H) Enrichment analysis of proteins from protein M3 (G) and M4 (H). The top five significantly enriched GO and KEGG pathways are displayed. The x-axis represents −log_10_[*P* values]. To filter for GO and KEGG enriched pathways, a significance threshold of *P*_FDR_ < 0.01 and < 0.05 was applied, respectively. The top five proteins with the largest intramodular connectivity and significant *P* value are displayed in the dashed box. Proteins marked with asterisks overlap with 381 identified proteins. Abbreviations: BP, biological processes; CC, cellular components; GO, Gene Ontology; KEGG, Kyoto Encyclopedia of Genes and Genomes; MF, molecular functions.

Furthermore, we investigated the plasma proteomic signatures of each subtype of childhood adversity (i.e., physical neglect, emotional neglect, sexual abuse, physical abuse, and emotional abuse), as shown in Fig. 1B. We further integrated five subtypes into three broader subtypes (i.e., emotional abuse and neglect, physical abuse and neglect, and sexual abuse) to investigate the plasma proteomic signatures (Fig. 1C). Notably, 10 proteins were identified as being associated with all three broader subtypes of adversity. Among them, apolipoprotein F (APOF) is involved in lipid metabolism, while C-X-C motif chemokine ligand 17 (CXCL17) plays a functional role in maintaining homeostasis at mucosal barriers. Moreover, growth differentiation factor 15 (GDF15), a key molecule in the mechanisms of human stress response, was found to be associated with both physical and emotional adversity. Notably, PIGR and GAST emerged as common proteins, consistently associated with all five subtypes, suggesting broad biological relevance.

In sex-stratified PWAS analyses, we identified 308 and 232 proteins that were significantly associated with childhood adversity in males and females, respectively (Tables S12 and S13). After FDR correction, only intercellular adhesion molecule 1 (ICAM1) remained significant in males, while GAST and PIGR were significant in females (Fig. S4). Notably, both GAST and PIGR were associated with all subtypes of childhood adversity in females, while no protein was consistently associated with all subtypes in males, which may arise from distinct immune responses to stress (Fig. S4).

To investigate the potential interactions between the identified proteins, we performed PPI analysis using the 381 proteins associated with childhood adversity. An interconnected network featuring 329 nodes and 1,906 edges was found (Fig. S5). Using the Analyze Network tool, we identified the top two hub immune-related proteins: protein tyrosine phosphatase receptor type C (PTPRC) and intercellular adhesion molecule 1 (ICAM1).

To elucidate the biological mechanisms underlying the 381 proteins’ association with childhood adversity, we performed Gene Ontology (GO) and Kyoto Encyclopedia of Genes and Genomes (KEGG) pathway enrichment analyses (Fig. 1D and 1E; Table S14). GO analysis identified significant enrichment in pathways including the external side of the plasma membrane, collagen-containing extracellular matrix, positive regulation of cell adhesion, leukocyte cell-cell adhesion, and regulation of T cell activation. Similarly, KEGG analysis revealed significant enrichment in pathways including cytokine-cytokine receptor interactions, cell adhesion molecules, and complement and coagulation cascades. These findings collectively underscore the central role of immune dysregulation in the biological embedding of childhood adversity.

Subtype-stratified pathway analyses revealed both shared and distinct biological signatures across childhood adversity subtypes (Fig. S6). Immune-related pathways, including collagen-containing extracellular matrix, leukocyte migration, chemotaxis, cell adhesion molecules, cytokine-cytokine receptor interactions, and complement and coagulation cascades were significantly enriched across three to five subtypes. Notably, sexual abuse exhibited unique associations with endocrine-related pathways, including the AGE-RAGE signaling pathway in diabetic complications and the relaxin signaling pathway. Physical abuse was linked to metabolism-related pathways, including tyrosine metabolism, phenylalanine metabolism and nucleotide metabolism. Physical neglect was associated with digestive system pathways, including pancreatic secretion and protein digestion and absorption. Emotional neglect and abuse were enriched in viral myocarditis and lipid and atherosclerosis, respectively, suggesting that emotional adversity may increase the risk of cardiovascular diseases.

Sex-stratified analyses further revealed both shared and sex-specific biological pathways associated with childhood adversity (Fig. S7). Shared pathways, including cytokine-cytokine receptor interaction and viral protein interaction with cytokine and cytokine receptor, were enriched in both sexes. In males, enriched pathways were mainly related to immune cell activation, proliferation, and signaling, including regulation of T cell activation, leukocyte proliferation, and PI3K-Akt signaling pathway, with additional enrichment in metabolic pathways such as thiamin metabolism and type I diabetes mellitus. In females, enriched pathways were predominantly associated with extracellular matrix remodeling, tissue injury response, and immune cell trafficking, including collagen-containing extracellular matrix, regulation of response to wounding, vesicle lumen, lysosomal lumen, leukocyte migration, and cell chemotaxis.

Of 2,919 proteins, nine protein modules (refer to M1–M9) were identified by conducting protein co-expression network analysis (Fig. S8). Based on the first principal component (PC) of each protein module, childhood adversity was positively associated with protein M3 (*P*_Bon_ = 0.013), M4 (*P*_Bon_ = 0.017) and M9 (*P*_Bon_ = 0.015) after Bonferroni correction (Figs. 1G, 1H and S8; Table S15). Pathway enrichment analysis revealed that these three protein modules were involved in distinct biological pathways. Specifically, M3 exhibited enrichment in pathways related to cellular immunity, including cytokine-cytokine receptor interaction, leukocyte cell-cell adhesion, regulation of T cell activation and regulation of lymphocyte proliferation (Fig. 1G). M4 demonstrated enrichment in metabolism-related pathways and included the top protein linked to childhood adversity, PIGR (Fig. 1H; Table S16). M9 showed enrichment in pathways related to cytokines, humoral immunity and complement system, including cytokine-cytokine receptor interaction, cytokine activity, humoral immune response, complement and coagulation cascades and complement activation, and included the second most strongly associated protein, GAST (Fig. S8; Table S16). Additionally, we observed that M9 was linked to PI3K-Akt and JAK-STAT signaling pathways (Fig. S8).

To investigate causal associations between childhood adversity and plasma proteomic alterations, we performed a unidirectional two-sample MR analysis. Using the inverse-variance weighted (IVW) method, we identified causal links between childhood adversity and 25 proteins (*P *< 0.05; Table S17), five of which overlapped with the 381 proteins identified in PWAS. Notably, only two proteins—PIGR and interleukin 17D (IL17D)—showed consistent positive associations in both PWAS and MR analyses (Fig. 2A; Tables S17 and S18) and were therefore selected for subsequent mediation analysis. IL17D functions as a key regulator of innate immune responses in inflammation and cancer. Sensitivity analyses further validated the robustness of these findings, as the results aligned with IVW estimates in direction. We found no evidence of heterogeneity (Q statistic, all *P *> 0.05) or horizontal pleiotropy (MR-Egger intercept and MR-PRESSO, all *P *> 0.05). Additionally, leave-one-out analyses confirmed that no single-nucleotide polymorphisms (SNPs) influenced the causal associations (Fig. S9; Table S19).

**Figure 2.**
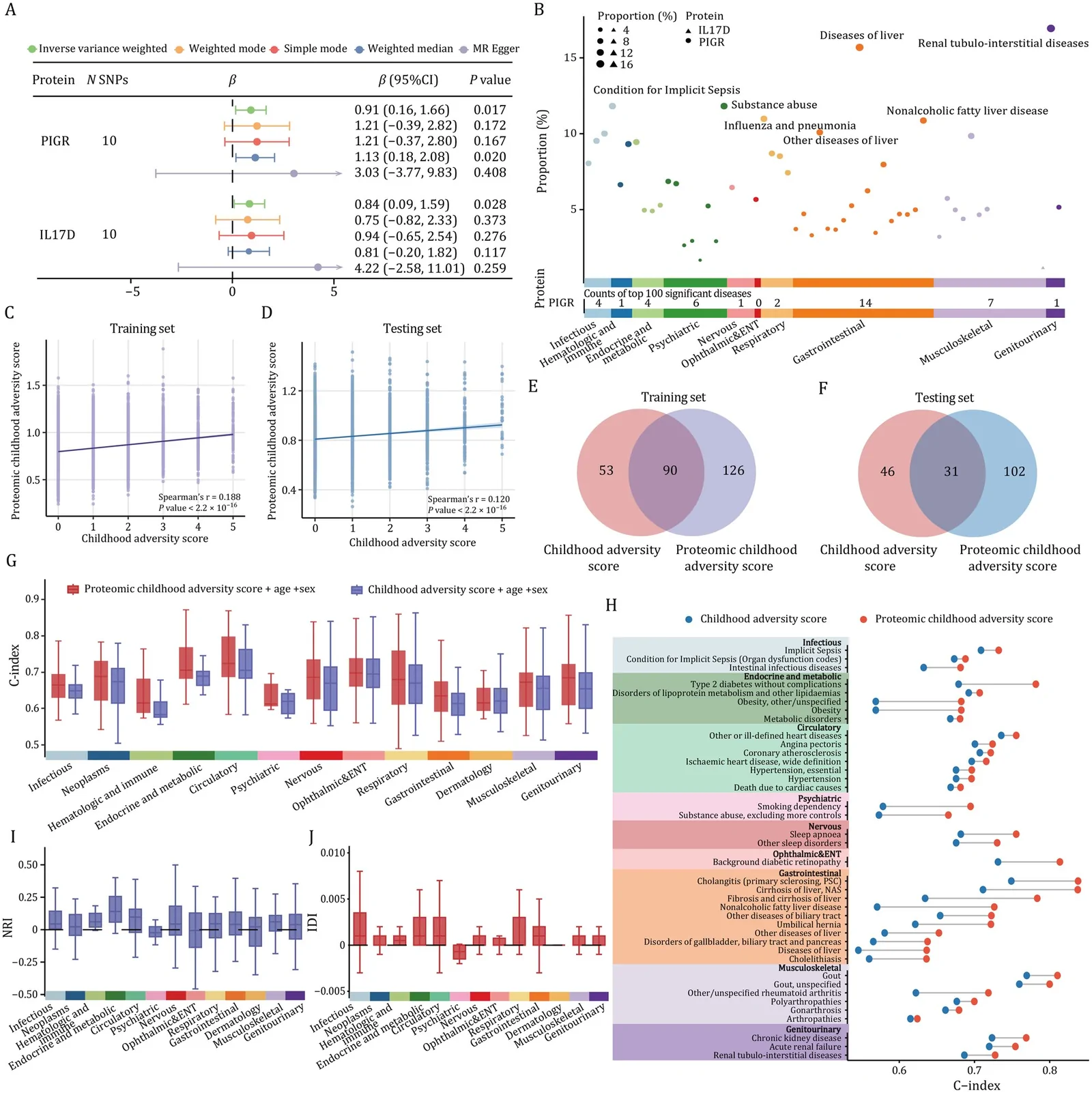
**Plasma proteins link childhood adversity to diseases**. (A) Summary of MR analysis on the associations between childhood adversity and proteins. The inverse variance weighted (IVW) method was used as the primary analytic approach, complemented by MR-Egger, simple mode, weighted median and weighted mode. A significance threshold of *P* < 0.05 was applied. Significant proteins identified by the IVW method, showing consistent positive directions of association in initial analyses, are presented. The dots represent β coefficients and the error bars indicate 95% CIs. (B) Mediating roles of two MR-identified proteins in the associations between childhood adversity and the risks of incident diseases. Linear regression models were performed to estimate the associations between childhood adversity and proteins. Cox proportional hazards models were performed to estimate the associations of childhood adversity and proteins with risks of incident diseases, respectively. All models were adjusted for age, sex, race and ethnicity, educational level, occupation, Townsend deprivation index, body mass index, smoking status, drinking status, and exercise status. Mediation analyses were conducted for the 51 incident diseases that showed significant associations with both childhood adversity and plasma proteins. The table below summarizes the counts of top 100 significant diseases for PIGR, adapted from Fig. S2B. Note that the sample size used in Fig. S2B differs from that used in the current analysis. IL17D is not shown, as it has no count among the top 100 significant diseases. The dots were colored by disease categories. The symbols represent different proteins, with colors indicating the kind of disease category. The height and size of each symbol in panel indicate the mediation proportion of proteins on the associations between childhood adversity and the risks of incident diseases. (C and D) Scatter plots showing the correlation between PCAS and traditional childhood adversity score in training (C) and testing set (D). Each point indicates a single participant. The Spearman’s correlation coefficient (*r*) is shown in the bottom right corner. (E and F) Venn diagrams showing the number of incident diseases associated with PCAS and traditional childhood adversity score and the overlaps among these associations (*P* < 0.05) in training (E) and testing set (F). (G) The discriminative performances for disease prediction assessed by the C-index across two models in testing set: PCAS + age + sex model, and childhood adversity score + age + sex model. The box plot shows the median (line), interquartile range (IQR) (box) and whiskers extending to 1.5 × IQR. (H) PCAS outperformed the traditional childhood adversity score in predicting 39 incident diseases across nine disease categories in testing set (*P *< 0.05). (I and J) The reclassification performances for disease prediction assessed by NRI (I) and IDI (J) across two models in testing set: PCAS + age + sex model, and childhood adversity score + age + sex model. The box plot shows the median (line), interquartile range (IQR) (box) and whiskers extending to 1.5 × IQR. Abbreviations: C-index, Harrell’s concordance index; ENT, ear, nose, and throat; IDI, integrated discrimination improvement; MR, Mendelian randomization; NRI, net reclassification improvement; SNP, single nucleotide polymorphism.

In addition, we investigated the mediating roles of PIGR and IL17D in the associations between childhood adversity and the risks of incident diseases (Fig. 2B; Tables S20–23). For each protein, mediation analyses were conducted for incident diseases that showed significant associations with both childhood adversity and the protein itself. PIGR was identified as a significant mediator in the associations between childhood adversity and 50 incident diseases, the majority of which fell within the gastrointestinal (17/50), psychiatric (8/50) and musculoskeletal (7/50) disease categories, with mediation proportions ranging from 1.7% to 16.9%. Notably, per 1-point increase in childhood adversity score, PIGR mediated the largest proportions of risk for the following three diseases: 16.9% (95% CI: 7.9%, 61.6%) for renal tubulo-interstitial diseases, 15.7% (95% CI: 7.1%, 61.3%) for liver diseases, and 11.8% (95% CI: 5.7%, 34.2%) for substance abuse. In contrast, IL17D showed a limited mediation effect, mediating 1.1% (95% CI: 0%, 4.1%) of the risks of fibroblastic disorders, which fell into the musculoskeletal disease category.

In analytic sample 2, participants were randomly partitioned into a training set (*N *= 9,053) and a testing set (*N *= 3,880) at a 7:3 ratio. Based on the 381 PWAS-significant plasma proteins, we constructed a PCAS in the training set using elastic net regression (the original model, hereafter) with 10-fold cross-validation (α = 0.2). The original model retained 114 proteins, including PIGR, GAST, APOE, GDF15 and others (Table S24). The Spearman correlation coefficients (*r*) between PCAS and the childhood adversity score were 0.188 and 0.120 in the training and testing sets, respectively (both *P *< 0.001; Fig. 2C and 2D). To rigorously assess model stability, we conducted a sensitivity analysis using a 10-fold cross-validation framework in which the entire analytical pipeline was independently repeated within each iteration (Supplementary Material). This procedure yielded 10 fold-specific sparse models, with the number of retained proteins ranging from 101 to 141. Importantly, a core set of 59 proteins was consistently selected across all 10 models, all of which were included in the original model (Fig. S10). To quantify robustness, we calculated pairwise Jaccard indices between all fold-specific models and the original model. The mean Jaccard similarity was 0.704, indicating substantial consistency in the selected protein sets despite the high dimensionality and correlation structure of the proteomic data (Fig. S10). Consistent with this feature-level stability, the PCAS derived from the 10 fold-specific models was highly correlated with that obtained from the original model (Pearson’s *r* = 0.970), supporting the robustness of the PCAS to resampling variation (Fig. S10). Functional enrichment analysis of the 114 proteins retained in the original model showed significant enrichment in pathways related to the external side of the plasma membrane, collagen-containing extracellular matrix, and cytokine activity, as well as KEGG pathways including integrin signaling, cytokine-cytokine receptor interaction, and folate transport and metabolism (Fig. S11). These results indicated that the PCAS proteins were primarily involved in immune-regulatory and extracellular matrix-related biological processes.

Tables S25–32 present the model performance for disease prediction in the training and testing sets. In the testing set, after adjusting for all covariates, PCAS was significantly associated with 133 incident diseases across 13 categories (*P *< 0.05), substantially more than the 77 diseases associated with self-reported childhood adversity score (Figs. 2F and S12; Tables S27 and S28). When integrated into a model including age and sex, the inclusion of PCAS yielded superior predictive accuracy compared to the self-reported childhood adversity score (Fig. 2G–J). Specifically, significant increments in Harrell’s concordance index (C-index) were observed for 39 incident diseases across nine categories (*P *< 0.05), with the most notable improvements clustered in gastrointestinal (10/39), circulatory (7/39), musculoskeletal (6/39), and endocrine and metabolic (5/39) disorders (Fig. 2G; Table S30). Notably, the greatest increases in C-index were observed for background nonalcoholic fatty liver disease (0.726 vs. 0.571), indicating markedly improved model discrimination when using PCAS (Fig. 2H; Table S30). The robust incremental value of the PCAS was further validated through clinical reclassification metrics. In the testing set, 82 and 102 incident diseases exhibited significant improvements in net reclassification improvement (NRI) and integrated discrimination improvement (IDI), respectively (*P *< 0.05). Crucially, 90.2% (74/82) of these significant NRIs and 82.4% (84/102) of the IDIs were positive, providing evidence that the PCAS enabled more precise and reliable disease risk stratification than conventional self-reported measures (Fig. 2I and 2J; Table S32). Furthermore, the predictive performance metrics in the training set were correlated with those in the testing set (Pearson’s *r* = 0.739 for C-index, 0.175 for NRI, and 0.693 for IDI; all *P *< 0.001) (Fig. S13).

In summary, this large-scale study, utilizing data from the UKB, comprehensively demonstrated that childhood adversity exhibited significant associations with 198 prevalent and 338 incident diseases, particularly psychiatric disorders, as well as musculoskeletal, gastrointestinal, respiratory and genitourinary diseases. We also identified 381 plasma proteins and three protein modules associated with childhood adversity, which were predominantly involved in biological pathways pertaining to immune function and metabolism. Moreover, we provided evidence suggesting that childhood adversity causally influenced the abundance of two proteins (i.e., PIGR and IL17D), which also mediated the associations between childhood adversity and the risk of 51 incident diseases, including gastrointestinal, psychiatric and musculoskeletal diseases. We further developed a novel proteome-based measure of childhood adversity—PCAS—which demonstrated improved performance over the self-reported score in predicting incident diseases, reflecting its ability to capture molecular alterations associated with early-life adversity. These findings offer new molecular insights into the proteomic mechanisms underlying the associations between childhood adversity and diseases and underscore the promising therapeutic value of plasma proteins for mitigating disease burdens among adults with a history of childhood adversity.

To the best of our knowledge, this study represents the first systematic investigation linking childhood adversity to 1,045 diseases across 14 categories, largely expanding the existing literature, with emphasis on mental health (e.g., mental disorders, mood disorders, depression, and anxiety). Moreover, our study substantially uncovers novel connections between childhood adversity and multisystem morbidity including musculoskeletal, gastrointestinal, respiratory, genitourinary diseases, and other diseases. The underlying mechanisms, which may involve impaired mitochondrial function in muscle (Duchowny et al., 2024), disrupted gut microbiota and brain-gut axis (Hantsoo et al., 2019), engagement in health-risk behaviors (e.g., smoking habits), and disturbance in bladder-gut-brain axis (Leue et al., 2017), warrant further investigation. Notably, sex-stratified analyses revealed that the associations were more pronounced in females than in males across a broad spectrum of diseases. The findings not only align with previous studies demonstrating stronger associations between traumatic experiences and depressive or anxiety symptoms in females and also add new evidence for sex disparities in other disease categories (e.g., musculoskeletal, gastrointestinal, respiratory, and genitourinary diseases).

This study provides the first comprehensive atlas of the plasma proteomic signatures of childhood adversity and offered new mechanistic insights into how early-life adversity may become biologically embedded. The pathway enrichment analysis demonstrated that the identified proteins associated with childhood adversity were primarily involved in immune function and metabolism, which extends our understanding of the potential molecular mechanisms underlying childhood adversity-health associations and suggests long-term immune-metabolic dysregulation as a key biological pathway. Of note, the cytokine-cytokine receptor interaction emerged as the most significantly enriched pathway among the identified proteins. Cytokines are essential for intercellular communications and immunoregulatory actions, and sustained cytokine dysregulation may represent an early biological imprint of childhood adversity that precedes the onset of stress-related psychiatric and cardiometabolic disorders. The complement and coagulation cascades were also identified as significant pathways, which are critical for immune surveillance and tissue homeostasis. Emerging evidence suggests that dysregulation of these systems contributes to psychosis and neuroinflammation and may become activated before the onset of clinical symptoms (Heurich et al., 2022). Additionally, we observed significant enrichment of PI3K-Akt signaling, a pathway central to cell survival, synaptic plasticity, and neurodegeneration, providing a potential molecular link between childhood adversity and long-term brain vulnerability. Moreover, glutathione metabolism, a key metabolic regulator of oxidative stress within protein M4, may contribute to neuronal injury and brain alterations following early-life adversity (Zalachoras et al., 2020), highlighting oxidative stress as another convergent biological mechanism underlying mental health disorders.

Intriguingly, our study revealed that distinct biological pathways were enriched across five subtypes of childhood adversity. Sexual abuse was primarily associated with endocrine-related pathways, consistent with long-term disruption of hypothalamic-pituitary-adrenal (HPA) function and stress hormone regulation (Rösler, 1994). Physical abuse was linked to metabolism-related pathways, suggesting impaired energy homeostasis and nutritional stress as dominant biological consequences. Physical neglect was enriched in digestive system-related pathways, potentially reflecting gut-immune interactions and chronic inflammatory responses to bodily injury. In contrast, emotional neglect and emotional abuse were mainly associated with cardiovascular-related pathways, aligning with epidemiological evidence connecting emotional stress to elevated cardiovascular risk (Vaccarino and Bremner, 2024). These findings highlight the subtype-specific impacts of childhood adversity on health and elucidate the proteomic-level biological mechanisms underlying the existing epidemiological associations between specific subtypes of childhood adversity and health outcomes (Ho et al., 2020). Thus, it is important to account for subtype-specific biological responses when designing personalized therapeutic interventions to mitigate the detrimental effects of childhood adversity, thereby enhancing their efficacy and precision.

In addition, our analyses suggest that the biological embedding of childhood adversity is modulated by sex. While both males and females exhibited evidence of immune dysregulation, males showed stronger proteomic signatures related to immune cell activation and proliferation, whereas females displayed more pronounced alterations in extracellular matrix remodeling, tissue injury response, and immune cell trafficking. These sex-specific proteomic patterns may help explain the broader and stronger associations observed in females across psychiatric, musculoskeletal, gastrointestinal, respiratory, and genitourinary diseases, likely reflecting sex-specific stress responses, immune reactivity, and HPA axis regulation influenced by gonadal hormone changes (Dunn et al., 2024). Together, these findings highlight the importance of considering sex-specific mechanisms when developing interventions aimed at mitigating the lifelong health consequences of childhood adversity.

Building on MR analysis findings, we further demonstrated that two plasma proteins—PIGR and IL17D—partially mediated the associations between childhood adversity and the risks of several incident diseases, particularly gastrointestinal, psychiatric and musculoskeletal diseases. While these proteins operate across distinct biological contexts, their roles converge on a shared mechanism of mucosal barrier-immune-brain axis dysregulation. PIGR, which can be influenced by stress hormones such as glucocorticoids, plays a critical role in regulating mucosal immunity. Additionally, studies suggest that the mRNA levels of PIGR rise slowly in response to cytokine stimulation (Kaetzel, 2005), which may explain its substantial mediating effects. Interleukin17 (IL17), an important cytokine, is critical for the proliferation and survival of fibroblastic reticular cells, which offers a possible mechanistic explanation for IL17D’s mediating role in the association between childhood adversity and fibroblastic disorders. Together, these findings suggest that childhood adversity may be associated with impaired mucosal immune regulation via PIGR, facilitating systemic immune activation, while IL17D-related stromal and immune remodeling may contribute to fibrotic and neuroinflammatory processes. Such cross-system interactions could help explain the increased risks across gastrointestinal, psychiatric, and musculoskeletal diseases observed in this study. These findings may lay the foundation for identifying therapeutic targets and advancing precision medicine approaches.

Interestingly, the newly developed PCAS demonstrated improved predictive performance compared with the self-reported childhood adversity score, indicating that the plasma proteome captures biological correlates of early-life adversity beyond questionnaire-based measures. Functional enrichment analyses showed that PCAS-related proteins were mainly involved in immune-regulatory and extracellular matrix-related pathways. Importantly, although PCAS is derived from childhood adversity data and therefore partially reflects biological imprinting of early-life adversity, several constituent proteins—such as APOE and GDF15—are also well known to be associated with adult lifestyle factors (including obesity, smoking, and high-fat dietary patterns). These proteins may therefore reflect the combined influence of early-life adversity and later social and behavioral environments across the life course. Thus, PCAS should not be interpreted as a childhood adversity-specific biomarker, but rather as a broader cumulative proteomic proxy integrating the long-term effects of childhood adversity with later life-course exposures. Overall, PCAS appears to capture a more general pattern of adversity-related biological embedding rather than childhood adversity in isolation.

In conclusion, this study represents the first comprehensive and systematic investigation of a broad spectrum of diseases and plasma proteomic signatures associated with childhood adversity. We identified significant and sex-specific associations between childhood adversity and multiple categories of diseases, particularly psychiatric disorders. More importantly, we identified 381 plasma proteins associated with childhood adversity, and two causal proteins served as significant mediators linking childhood adversity to several diseases, pointing to the crucial role of biological pathways pertaining to immune function and metabolism. Furthermore, we developed PCAS, a novel proteome-based measure of childhood adversity, which exhibited enhanced predictive performance for incident diseases, suggesting it reflects general patterns of biological embedding associated with adversity across the life course. Despite multiple testing corrections, potential false positives remain possible given the large-scale analysis, and results warrant cautious interpretation and independent validation. Although the proteomics subsample was broadly representative, its relative overrepresentation of White and socioeconomically advantaged participants may limit generalizability to more diverse or disadvantaged populations. Overall, these findings underscore the potential of plasma proteins in elucidating the connections between childhood adversity and multiple diseases, thereby paving the way for more personalized and effective therapeutic strategies for adults who have experienced childhood adversity.

## Supplementary Material

pwag028_Supplementary_Data

## Footnotes

This research has been conducted using the UK Biobank resource under application number 61856. We wish to acknowledge the UK Biobank participants who provided the sample that made the data available.

This research was supported by grants from the National Natural Science Foundation of China (72374180), Research Center of Prevention and Treatment of Senescence Syndrome, School of Medicine Zhejiang University (2022010002), “Pioneer” and “Leading Goose” R&D Programs of Zhejiang Province (2025C02104), Zhejiang Key Laboratory of Intelligent Preventive Medicine, and Economic and Social Research Council & Biotechnology & Biological Sciences Research Council (UK), ES/T00200X/1 (2765580, 2765592). The funders had no role in the study design, data collection, analysis, or interpretation; in the writing of the report; or in the decision to submit the article for publication.

Jiening Yu, Duoduo Fu, Xucheng Wu, Weijing Gao, Hongwei Chen, Xueqing Jia, Fan Pu, Yanjie Zhao, Kaili Sun, Liming Zhang, Wen Wang, Huiqian Huang, Anna Dearman, and Zuyun Liu declare that they have no conflict of interest.

Ethics approval and consent to participate UK Biobank has approval from the North West Multi-Centre Research Ethics Committee as a Research Tissue Bank approval in 2011 and is renewed every 5 years, which allowed researchers to use data from UK Biobank without an additional ethical clearance. All participants have provided signed informed consent.

The authors declare their agreement to participate. The authors declare their agreement to publish.

The data that support the findings of this study are openly available in UK Biobank at (ukbiobank.ac.uk/), reference number [61856].

The original code used in the study is available from Zuyun Liu (Zuyun.liu@outlook.com or zuyunliu@zju.edu.cn) upon request.

Z.L. designed and supervised the study. J.Y., D.F., W.G., X.J., F.P., and Y.Z. contributed to data collection. J.Y., D.F., W.G., H.C., and F.P. analyzed the data. J.Y., D.F., W.G., and F.P. interpreted the results. J.Y., D.F., X.W., and W.G. drafted the manuscript. J.Y., D.F., and X.W. visualized the results. X.J, K.S, L.Z., W.W., H.H., and A.D. contributed to the interpretation of findings. All authors revised the manuscript. Z.L. and J.Y. had full access to all the data in the study. Z.L. took responsibility for the accuracy of the data analysis and the content of the article. All authors read and approved the submitted version of the manuscript. No AI tools were used in this work.
