# Supplementary material for: Plasma proteomic signatures of childhood adversity
Source: Protein Cell. 2026 Apr 22;17(10):987–95. doi: 10.1093/procel/pwag028 (PMC13626735; doi:10.1093/procel/pwag028)
Supplement: Supplementary file 1 [file pwag028_supplementary_data.zip › Supplementary_Methods_and_Figures_S1-S13.docx]

**Supplementary materials for**

**Plasma proteomic signatures of childhood adversity**

Jiening Yu^1^, Duoduo Fu^2^, Xucheng Wu^1^, Weijing Gao^1^, Hongwei Chen^1^, Fan Pu^1^, Yanjie Zhao^1^, Kaili Sun^1^, Liming Zhang^1,3^, Wen Wang^4^, Huiqian Huang^5^, Anna Dearman^4^, Zuyun Liu^1*^

^1^ Center for Clinical Big Data and Analytics Second Affiliated Hospital, Department of Big Data in Health Science School of Public Health, Zhejiang Key Laboratory of Intelligent Preventive Medicine, Zhejiang University School of Medicine, Hangzhou 310058, Zhejiang, China

^2^ Department of Epidemiology, Harvard T.H. Chan School of Public Health, Boston, MA 02115, USA

^3^ Department of Health Policy and Management, Yale School of Public Health, New Haven, CT 06520, USA

^4^ Institute for Social and Economic Research, University of Essex, Wivenhoe Park, Colchester, Essex, CO4 3SQ, UK

^5^ Basic and Translational Research Center, The Second Affiliated Hospital, Zhejiang University School of Medicine, Hangzhou 310058, Zhejiang, China

***Corresponding author:** Zuyun Liu, Professor, Center for Clinical Big Data and Analytics Second Affiliated Hospital, Department of Big Data in Health Science School of Public Health, Zhejiang Key Laboratory of Intelligent Preventive Medicine, Zhejiang University School of Medicine, 866 Yuhangtang Rd, Hangzhou 310058, Zhejiang, China. Telephone: +86-0571-87077127.

E-mail: Zuyun.liu@outlook.com or [zuyunliu@zju.edu.cn](mailto:zuyunliu@zju.edu.cn)

**Materials and Methods**

**Figure S1.** Flow chart of the analytic sample

**Figure S2.** Mapping 1,045 diseases to childhood adversity in 150,147 individuals

**Figure S3.** Sex-stratified analyses and sensitivity analyses of diseases associated with childhood adversity

**Figure S4.** Sex-stratified analyses of associations between childhood adversity and proteins

**Figure S5.** Protein-protein interaction network (PPI) analysis for the 381 identified proteins

**Figure S6.** Results of pathway enrichment analysis for five subtypes of childhood adversity

**Figure S7.** Results of sex-specific pathway enrichment analyses

**Figure S8.** Associations between childhood adversity and protein modules among 12,933 individuals

**Figure S9.** Results of leave-one-out sensitivity analysis for MR using IVW method

**Figure S10.** Stability assessment of proteomic childhood adversity score (PCAS)

**Figure S11.** Results of pathway enrichment analyses for proteins selected to construct proteomic childhood adversity score (PCAS).

**Figure S12.** Associations between childhood adversity score, proteomic childhood adversity score (PCAS) and incident diseases in training and testing sets

**Figure S13. Correlation of performance metrics between the training and testing sets**

**Table S1.** Baseline characteristics of the analytic sample

**Table S2.** Baseline characteristics comparison between the analytic sample 1 and 2

**Table S3.** Associations of childhood adversity with 396 prevalent diseases in analytic sample 1 (N = 150,147)

**Table S4.** Associations of childhood adversity with 649 incident diseases in analytic sample 1 (N = 150,147)

**Table S5.** Associations of childhood adversity with 334 prevalent diseases in male of analytic sample 1 (N = 65,613)

**Table S6.** Associations of childhood adversity with 388 prevalent diseases in female of analytic sample 1 (N = 84,534)

**Table S7.** Associations of childhood adversity with 620 incident diseases in male of analytic sample 1 (N = 65,613)

**Table S8.** Associations of childhood adversity with 638 incident diseases in female of analytic sample 1 (N = 84,534)

**Table S9.** Sensitivity analyses of associations of childhood adversity with 396 prevalent diseases in analytic sample 1 (N = 150,147)

**Table S10.** Sensitivity analyses of associations of childhood adversity with 649 incident diseases in analytic sample 1 (N = 150,147)

**Table S11.** Significant results of proteome-wide association study for childhood adversity in analytic sample 2 (N = 12,933)

**Table S12.** Significant results of proteome-wide association study for childhood adversity in male of analytic sample 2 (N = 5,708)

**Table S13.** Significant results of proteome-wide association study for childhood adversity in female of analytic sample 2 (N = 7,225)

**Table S14.** Significant pathway enrichments in proteins associated with childhood adversity

**Table S15.** Associations of childhood adversity with protein modules

**Table S16.** Proteins in module 3, 4, and 9 and their correlations to the module eigengene.

**Table S17.** Significant results of MR analyses on the associations between childhood adversity and proteins.

**Table S18.** SNPs used in MR analyses of the associations between childhood adversity and PIGR and IL17D.

**Table S19.** MR sensitivity analysis of associations between childhood adversity and PIGR and IL17D.

**Table S20.** Associations of childhood adversity with 645 incident diseases in analytic sample 2 (N =12,933)

**Table S21.** Associations of PIGR with 645 incident diseases in analytic sample 2 (N =12,933)

**Table S22.** Associations of IL17D with 645 incident diseases in analytic sample 2 (N =12,933)

**Table S23.** Mediation role of PIGR and IL17D on the associations between childhood adversity and incident diseases

**Table S24.** Weights of the proteins included in the proteomic childhood adversity score derived using elastic net regression in training set (N =9,053)

**Table S25.** Associations of childhood adversity with 638 incident diseases in training set (N = 9,053)

**Table S26.** Associations of proteomic childhood adversity score with 638 incident diseases in training set (N = 9,053)

**Table S27.** Associations of childhood adversity with 496 incident diseases in testing set (N = 3,880)

**Table S28.** Associations of proteomic childhood adversity score with 638 incident diseases in testing set (N = 3,880)

**Table S29.** C-index of model for predicting 645 incident diseases in training set (N =9,053)

**Table S30.** C-index of model for predicting 645 incident diseases in testing set (N =3,880)

**Table S31.** NRI and IDI of model for predicting 645 incident diseases in training set (N =9,053)

**Table S32.** NRI and IDI of model for predicting 495 incident diseases in testing set (N =3,880)

**Table S33.** Questions and responses for variables included in the childhood adversity in UK Biobank

**Table S34.** Characteristics and definition of prevalent diseases

**Table S35.** Characteristics and definition of incident diseases

**Materials and Methods**

**Study population**

The UKB is a large-scale prospective cohort study that enrolled over 500,000 participants aged 40-69 years through 22 assessment centers across England, Scotland, and Wales recruited between 2006 and 2010. Baseline data collection incorporated self-reported questionnaires, physical measurements, biological samples, and whole-genome genotyping. A comprehensive description of the study design and methodology has been provided elsewhere (Sudlow et al., 2015). The study protocol was approved by the North West Multi-Centre Research Ethics Committee, and all participants provided informed consent. In 2016, approximately one-third of the participants (*N* = 156,749) responded to the online mental health questionnaire including five-item Childhood Trauma Screener for assessing childhood adversity. Participants with missing data on childhood adversity (*N* = 3,728) and covariates (*N* = 2,874) were excluded, resulting in an analytic sample 1 of 150,147 participants for analysis between childhood adversity and diseases. After further excluding the participants with missing data on plasma proteomics (*N* = 137,214), an analytic sample 2 of 12,933 participants remained for investigating the plasma proteomic signatures of childhood adversity (**Fig. S1**). The median follow-up duration was 13.8 years (ending on death, loss to follow-up, or the date of censoring [October 31, 2022]).

**Childhood adversity**

Childhood adversity was evaluated using Childhood Trauma Screener, a cost-efficient and validated screening tool, as part of the 2016 online mental health questionnaire. Childhood Trauma Screener evaluated five items, including physical neglect, emotional neglect, sexual abuse, physical abuse, and emotional abuse (Glaesmer et al., 2013): 1) someone to take to doctor when needed as a child (physical neglect); 2) felt loved as a child (emotional neglect); 3) sexually molested as a child (sexual abuse); 4) physically abused by family as a child (physical abuse); and 5) felt hated by a family member (emotional abuse) (Grabe et al., 2012). Each item was introduced with the phrase: “When I was growing up (age <16 years old)” and rated on a five-point scale: never true, rarely true, sometimes true, often true, and very often true. Responses were dichotomized according to validated cutoff points from previous studies (Glaesmer et al., 2013, Soares et al., 2020). Physical neglect was assigned as 1 if participants reported never true, rarely true, sometimes true, or often true; emotional neglect was assigned as 1 if participants reported never true, rarely true, or sometimes true; sexual abuse, physical abuse, and emotional abuse was assigned as 1 if participants reported rarely true, sometimes true, often true, and very often true. The final self-reported childhood adversity score ranged from 0 to 5, with higher scores indicating greater exposure to childhood adversity. The details of questions and cutoff points are shown in **Table S33**.

**Disease definition**

Diseases were ascertained through linkage with UK electronic health records, utilizing International Classification of Diseases (ICD)-10 codes extracted from hospital inpatient records (category 2000, fields 41270 and 41280). Disease events were classified as prevalent (occurring before baseline visit) or incident (occurring after baseline visit), with the latter analyzed as time-to-event data. Participants at baseline with a prior diagnosis of a given disease were excluded from its corresponding incident disease analysis. Follow-up duration spanned from baseline visit until disease diagnosis, death, loss to follow-up, or the date of censoring (October 31, 2022), whichever came first.

Disease endpoints were defined following FinnGen criteria (see details in https://www.finngen.fi/), incorporating pre-defined quality control protocols for sex- or age-specific conditions and particular disease. The final analytical dataset comprised 655 incident and 404 prevalent disease endpoints across 14 disease categories according to a previous study (Deng et al., 2025). Detailed disease definitions and quality control criteria can be found in **Tables S34 and S35**.

**Plasma proteomics**

The UKB-PPP performed large-scale plasma proteomic profiling using the Olink Explore™ Proximity Extension Assay and next-generation sequencing (Wik et al., 2021). Following stringent quality control procedures (see details in https://biobank.ndph.ox.ac.uk/ukb/ukb/docs/PPP_Phase_1_QC_dataset_companion_doc.pdf), the high-throughput approach quantified 2,923 unique proteins across four panels covering cardiometabolic, inflammation, neurology and oncology. To mitigate batch effects, technical variabilities and enhance the measurement accuracy of low-abundance proteins, raw protein values were transformed to normalized protein expression (NPX), an arbitrary unit on a log^2^ scale (Sun et al., 2023, Wik et al., 2021). To reduce bias from excessive missingness, proteins with more than 20% missing NPX values were excluded, resulting in 2,919 proteins for analysis. Remaining missing values were imputed using a k-nearest neighbor (KNN) method (k = 10, Euclidean distance) implemented in the ‘impute’ R package (v1.80.0) (Fix and Hodges, 1989), with the protein matrix transposed as required. This approach preserves the correlation structure of high-dimensional proteomic data. No additional outlier filtering was performed beyond the standard quality control procedures of the UKB Olink proteomics pipeline.

**Covariates**

Covariate selection was guided by a priori complicated considerations, including age, sex, race and ethnicity, educational level, occupation, Townsend Deprivation Index (TDI), body mass index (BMI), smoking status, drinking status and exercise status, which were collected at baseline. Sociodemographic characteristics (i.e. age, sex, race and ethnicity, educational level, occupation, TDI) were treated as established confounders of the childhood adversity-outcome association. Lifestyle factors (i.e., BMI, smoking status, drinking status and exercise status) were included as potential confounders rather than mediators because they were shaped by shared upstream determinants associated with both childhood adversity and disease outcomes as well as proteomic profiles, and may also influence the reporting of childhood adversity. Race and ethnicity were classified as White, Mixed, South Asian, Black, Chinese, or other (Cao et al., 2020). Educational level was classified as high (college or university degree), intermediate (A/AS levels or O-levels, general certificate of secondary education, or equivalent), and low (none of the above) (Zhang et al., 2021). Occupation was classified as working, retired, or other (unpaid or voluntary work, full-time or part-time student, looking after home and/or family, unable to work because of sickness or disability, unemployed, or did not answer) (Chudasama et al., 2020). 2011 Census data on employment, car ownership, home ownership, and overcrowding based on the postal code of participants was used for TDI. A higher TDI indicates a higher area-level of material deprivation. BMI was calculated as body weight/height^2^ in kg/m^2^. Smoking and drinking status were both self-reported and classified as never, former, and current use. Exercise status was defined as regular exercise if participants met the global health recommendations for physical activity per week (150 minutes of moderate activity or 75 minutes of vigorous activity or an equivalent combination) (Chudasama et al., 2020). Otherwise, it was classified as non-regular exercise.

**Statistical analysis**

**Associations of childhood adversity with diseases.** In analytic sample 1, we excluded disease endpoints with fewer than 100 cases, resulting in a total of 649 incident and 396 prevalent disease endpoints. For prevalent diseases, logistic regression models were performed to investigate the associations of childhood adversity with prevalent diseases, adjusted for the covariates listed above. For incident diseases, Cox proportional hazards models were performed to investigate the associations of childhood adversity with the risks of incident disease, adjusted for the covariates listed above. Multiple testing correction was applied using the Benjamini-Hochberg FDR method. The corresponding *P* values and ORs (or HRs) for all associations were documented.

In addition, sex-stratified analyses were conducted to provide further insights. Moreover, sensitivity analyses were performed to investigate the influence of comorbidity based on evidence from a recent study (Deng et al., 2025). We defined 14 binary variables of multimorbidity based on 14 disease categories at baseline (**Tables S34 and S35**). For prevalent diseases, participants diagnosed with any disease from the same category were excluded from the control group, and models were adjusted for 13 binary variables of other disease categories. For incident diseases, participants diagnosed before baseline were excluded from the case group, and those with incident diseases were excluded from the control group, with models adjusted for 12 binary variables of other disease categories.

**Proteins associated with childhood adversity.** In analytic sample 2, PWAS was performed using linear regression models to evaluate associations between childhood adversity and protein levels, adjusting for all covariates. Additional analyses evaluated the associations between each individual item of childhood adversity (i.e., physical neglect, emotional neglect, sexual abuse, physical abuse, and emotional abuse) and protein levels. Moreover, sex-stratified analyses were conducted.

**PPI network analysis.** In analytic sample 2, the PPI network of the identified proteins associated with childhood adversity was constructed using STRING with default settings and visualized by Cytoscape. The Analyze Network tool in Cytoscape was utilized to identify hub proteins.

**Protein co-expression network analysis.** In analytic sample 2, gene co-expression network analysis (WGCNA) was implemented to identify co-expression protein modules using the ‘WGCNA’ R package, with the full set of 2,919 proteins from 12,933 participants. We aggregated information in the protein modules using the first PC. Furthermore, the top five proteins with the largest intramodular connectivity and significant *P* values were defined as the hub proteins of each protein module. The associations between childhood adversity and protein modules were evaluated using linear regression models, adjusting for all covariates listed above. The *P* values were corrected using the Bonferroni method.

**Pathway enrichment analysis.** In analytic sample 2, GO and KEGG pathway analyses were performed using the ‘clusterProfiler’ R package to investigate the potential biological pathways associated with the identified proteins or protein modules linked to childhood adversity. GO pathway analysis was conducted across three domains: cellular components (CC), biological processes (BP), and molecular functions (MF). KEGG pathway analysis was employed to identify relevant functional pathways. To filter for GO and KEGG enriched pathways, a significance threshold of *P* < 0.01 and < 0.05 after FDR correction were applied, respectively.

**MR.** We applied unidirectional two-sample MR to investigate the causal relationship between childhood adversity and identified proteins. The genome-wide association studies (GWAS) summary statistics for childhood adversity were derived from 185,414 participants of European ancestry across five cohorts: the UKB, Adolescent Brain Cognitive Development Study, Avon Longitudinal Study of Parents and Children, Generation R Study, and Psychiatric Genomics Consortium. These data were sourced from a previously published large-scale genome-wide meta-analysis and are available for download at <https://doi.org/10.17863/CAM.65339>(Warrier et al., 2021). Protein Quantitative Trait Loci (pQTL) are genetic variations or loci associated with changes in protein levels. Similar to expression quantitative trait loci (eQTLs), pQTLs represent genomic positions linked to specific protein expression levels in specific tissues. The primary sources of protein GWAS data for the European population are the following studies: Sun et al. (3,283 proteins, *N* = 3,301; GWAS ID: prot-a-2235) (Sun et al., 2018), Folkersen et al. (83 proteins, *N* = 3,394; GWAS ID: prot-b-33) (Folkersen et al., 2017), and Suhre et al. (1,124 proteins, *N* = 997; GWAS ID: prot-c-3341_33_4) (Suhre et al., 2017). All relevant protein GWAS data can be accessed at <https://gwas.mrcieu.ac.uk/>. The GWAS summary statistics for childhood adversity were used for the selection of instruments. The following criteria were applied for selecting instruments: (i) SNPs associated with any protein were selected (*P* < 5×10^-8^); (ii) linkage disequilibrium clumping was performed to identify independent SNPs (r² < 0.001); and (iii) the R^2^ and F-statistic were used to estimate the strength of genetic instruments, where R^2^ was the proportion of the variability of protein levels explained by each genetic instrument, and SNPs with an F-statistic less than 10 were excluded. The ‘TwoSampleMR’ R package was used to perform the MR analysis. The IVW method was applied to obtain MR effect estimates for proteins with more than one instrument. A heterogeneity test was performed to assess the heterogeneity of the genetic instruments using the Q statistic. MR-Egger was conducted to account for horizontal pleiotropy. MR-PRESSO was used as an additional method to detect and correct for outliers and distortions in the MR analysis. Sensitivity analyses were performed using simple mode, weighted mode, weighted median and MR-Egger methods. A leave-one-out analysis was performed to assess the influence of individual SNPs on MR estimation. Considering that childhood adversity occurred in early life (before age 18), whereas plasma protein levels were measured in adulthood, we did not perform directionality tests (e.g., Steiger filtering) in this study.

**Mediation analysis.** In analytic sample 2, we excluded disease endpoints with fewer than 10 cases, resulting in a total of 645 incident disease endpoints. The proteins that showed consistent associations with childhood adversity in both the PWAS and the MR analyses were selected as potential mediators. First, Cox proportional hazards models were employed to estimate the associations between childhood adversity and risks of incident diseases, adjusting for all covariates (**Table S20**). Second, the associations of each selected protein with risks of incident diseases were evaluated using Cox proportional hazards regression, adjusting for all covariates (**Tables S21-S22**). *P* values were adjusted using the Benjamini-Hochberg FDR method. Third, for each selected protein, we performed mediation analyses using the ‘mediation’ R package for those diseases simultaneously associated with childhood adversity and protein.

**Development of PCAS.** In analytic sample 2, we randomly partitioned the participants into a training set (*N* = 9,053) and a testing set (*N* = 3,880) using a 7:3 ratio. Within the training set, the PCAS was derived using the elastic net regression with 10-fold cross-validation (‘glmnet’ R package). The childhood adversity score was regressed on the significant plasma proteins previously identified through PWAS (original model). The mixing parameter (α) was selected through a grid search ranging from 0.1 to 0.9, and the regularization parameter (λ) was determined by cross-validation. PCAS values were calculated as a weighted sum of the selected proteins, with weights corresponding to the coefficients from the original elastic net model. Spearman’s correlation coefficients (r) between PCAS and self-reported childhood adversity score were calculated in both the training and testing sets. To assess the stability of protein selection, participants in the training set were further randomly partitioned into ten folds. In each iteration, one fold served as the testing (hold-out) set, and the remaining nine folds were used for model training. All data processing procedures, including feature selection and model fitting, were conducted exclusively within the training data of each iteration. This procedure was repeated ten times to generate ten fold-specific sparse models and corresponding predictions for each hold-out set. To further quantify robustness, the selection frequencies of individual proteins across the ten models were summarized to identify a set of consistently selected core proteins. Feature selection stability was additionally evaluated by calculating pairwise Jaccard indices among the protein sets identified in the ten fold-specific models and the original model. In addition, Pearson’s correlation coefficient (r) was computed to assess the agreement between PCAS derived from the fold-specific models and that derived from the original model. After excluding diseases with fewer than 10 incident cases, Cox proportional hazards models were used to estimate associations between PCAS and risks of 638 and 496 incident diseases in the training and testing sets, respectively, with adjustment for covariates. Predictive performance was evaluated using Harrell’s C-index (‘compareC’ R package), net reclassification improvement (NRI), and integrated discrimination improvement (IDI) (‘survIDINRI’ R package), comparing models including age, sex, and PCAS with models using the self-reported childhood adversity score. To examine the consistency of model performance across datasets, Pearson’s correlation coefficients (r) were calculated for each performance metric between the training and testing sets. *P* values were adjusted using the Benjamini-Hochberg FDR method.

All analyses were performed in R 4.4.2 (http://www.r-project.org) with a two-tailed *P* < 0.05 considered statistically significant unless noted.

**Figure S1. Flow chart of the analytic sample.** UKB, UK Biobank.

**Figure S2. Mapping 1,045 diseases to childhood adversity in 150,147 individuals**

(A) Associations between childhood adversity and 396 prevalent diseases revealed by logistic regression models. (B) Associations between childhood adversity and 649 incident diseases revealed by Cox proportional hazards models. All models were adjusted for age, sex, race and ethnicity, educational level, occupation, Townsend Deprivation Index, body mass index, smoking status, drinking status and exercise status. The dots were colored by disease categories. Filled dots refer to positive associations (odds ratios [ORs] or hazard ratios [HRs] > 1), while open dots indicate negative associations (ORs or HRs < 1). The y-axis represents −log_10_ (*P* values). A significance threshold of *P*_FDR_ < 0.05 was applied. ENT, ear, nose, and throat.

**Figure S3. Sex-stratified analyses and sensitivity analyses of diseases associated with childhood adversity**

(A-B) Associations between childhood adversity and 334 and 388 prevalent diseases in male (A) and female (B) revealed by logistic regression models, respectively. (C-D) Associations between childhood adversity and 620 and 638 incident diseases in male (C) and female (D) revealed by Cox proportional hazards models, respectively. In sex-stratified analyses, all models were adjusted for age, race and ethnicity, educational level, occupation, Townsend Deprivation Index, body mass index, smoking status, drinking status and exercise status. (E-F) Associations between childhood adversity and 396 prevalent diseases (E) and 649 incident diseases (F) in sensitivity analyses. For prevalent diseases, participants diagnosed with any disease from the same category were excluded from the control group, and models were adjusted for 13 binary variables of other disease categories. For incident diseases, participants diagnosed before baseline were excluded from the case group, and those with incident diseases were excluded from the control group, with models adjusted for 12 binary variables of other disease categories. The dots were colored by disease categories. Filled dots refer to positive associations (odds ratios [ORs] or hazard ratios [HRs] > 1), while open dots indicate negative associations (ORs or HRs < 1). The y-axis represents −log10 (*P* values). A significance threshold of *P*_FDR_ < 0.05 was applied. ENT, ear, nose, and throat.

**Figure S4. Sex-stratified analyses of associations between childhood adversity and proteins**

(A-B) Associations of childhood adversity with protein levels in male (A) and female (B), respectively. Linear regression models were performed, adjusted for age, race and ethnicity, educational level, occupation, Townsend Deprivation Index, body mass index, smoking status, drinking status and exercise status. The x-axis represents βs, and the y-axis represents −log_10_ (*P* values). The pie charts depict the proportions of 308 and 232 identified proteins across four distinct panels in male and female, respectively. (C-D) The overlapping of proteins across five subtypes of childhood adversity (i.e., physical neglect, emotional neglect, sexual abuse, physical abuse, and emotional abuse) in male (C) and female (D), respectively. The y-axis represents the number of overlapping proteins identified for each combination of subtypes (intersection size). The horizontal bars on the left display the total number of proteins associated with each subtype individually (set size). The dots and connecting lines indicate specific subtype combinations, with vertical bars showing the number of proteins shared among those subtypes. No proteins were simultaneously associated with five subtypes in male. PIGR and GAST were simultaneously associated with five subtypes in female.

**Figure S5. Protein-protein interaction network (PPI) analysis for the 381 identified proteins.**

The node size reflects the degree estimated by the Analyze Network tool and line thickness represents the combined score.

**Figure S6. Results of pathway enrichment analysis for five subtypes of childhood adversity**

(A-E) The top 10 enriched GO cellular components, biological processes and molecular functions for proteins linked to sexual abuse (A), physical neglect (B), physical abuse (C), emotional neglect (D) and emotional abuse (E), respectively. (F-J) The top 10 enriched KEGG pathways for proteins linked to sexual abuse (F), physical neglect (G), physical abuse (H), emotional neglect (I) and emotional abuse (J), respectively. For sexual abuse and emotional neglect, the number of significantly enriched KEGG pathways was fewer than 10. The x-axis represents −log_10_ (*P* values). To filter for GO and KEGG enriched pathways, a significance threshold of *P*_FDR_ < 0.01 and < 0.05 was applied, respectively. GO, Gene Ontology; KEGG, Kyoto Encyclopedia of Genes and Genomes.

**Figure S7. Result of sex-specific pathway enrichment analyses.**

(A) The top 10 enriched GO cellular components, biological processes and molecular functions for proteins identified in male. (B) The top 10 enriched KEGG pathways for proteins identified in male. (C) The top 10 enriched GO cellular components, biological processes and molecular functions for proteins identified in female. (D) The top 10 enriched KEGG pathways for proteins identified in female. For female, the number of significantly enriched KEGG pathways was fewer than 10. The x-axis represents −log_10_ (*P* values). To filter for GO and KEGG enriched pathways, a significance threshold of *P*_FDR_ < 0.01 and < 0.05 was applied, respectively. GO, Gene Ontology; KEGG, Kyoto Encyclopedia of Genes and Genomes.

**Figure S8. Associations between childhood adversity and protein modules among 12,933 individuals**

(A) Hierarchical cluster tree of 2,919 proteins measured at baseline. The band displays the separation of proteins into nine modules (refer to M1-M9) through weighted gene co-expression network analysis. (B) Hierarchical cluster tree of nine protein modules. (C) Enrichment analysis of proteins from protein M9. The top five significantly enriched GO and KEGG pathways are displayed. *P* values were adjusted using the Benjamini-Hochberg FDR method. The x-axis represents −log_10_ (*P* values). The top five proteins with the largest intramodular connectivity and significant *P* value are displayed in the dashed box. Proteins marked with asterisks overlap with 381 identified proteins. GO, Gene Ontology; KEGG, Kyoto Encyclopedia of Genes and Genomes; CC, cellular components; BP, biological processes; MF, molecular functions.

**Figure S9. Results of leave-one-out sensitivity analysis for MR using IVW method**

(A) Leave-one-out IVW analyses of the MR between childhood adversity and PIGR. (B) Leave-one-out IVW analyses of the MR between childhood adversity and IL17D. Dots represent MR effect size, and error bars represent 95% confidence intervals. The y-axis displays the excluded genetic variant per MR estimate. MR, Mendelian randomization; IVW, inverse-variance weighted.

**Figure S10. Stability assessment of proteomic childhood adversity score (PCAS).**

(A) Distribution of protein selection frequencies across the 10 fold-specific models. (B) Heatmap of pairwise Jaccard similarity indices between the sets of proteins selected in each of the ten folds and the original model. Higher values indicate greater overlap in feature selection across 11 models. (C) Scatter plot showing the correlations of the PCAS constructed from 10 fold-specific models with the PCAS constructed from the original model in training set (*N* = 9,053). Each point represents a single participant.

**Figure S11. Results of pathway enrichment analyses for proteins selected to construct proteomic childhood adversity score (PCAS).**

(A) The top 10 enriched GO cellular components, biological processes and molecular functions for 114 proteins selected to construct the PCAS. (B) The top 10 enriched KEGG pathways for 114 proteins selected to construct the PCAS. The number of significantly enriched KEGG pathways was fewer than 10. The x-axis represents −log_10_ (*P* values). To filter for GO and KEGG enriched pathways, a significance threshold of *P*_FDR_ < 0.01 and < 0.10 was applied, respectively. GO, Gene Ontology; KEGG, Kyoto Encyclopedia of Genes and Genomes.

**Figure S12. Associations between childhood adversity score, proteomic childhood adversity score (PCAS) and incident diseases in training and testing sets.**

(A-B) Associations between childhood adversity score (A), PCAS (B) and 638 incident diseases in training set (N = 9,053). (C-D) Associations between childhood adversity score (C), PCAS (D) and 496 incident diseases in testing set (N = 3,880). All models were adjusted for age, sex, race and ethnicity, educational level, occupation, Townsend Deprivation Index, body mass index, smoking status, drinking status and exercise status. The dots were colored by disease categories. Filled dots refer to positive associations (hazard ratios [HRs] > 1), while open dots indicate negative associations (HRs < 1). The y-axis represents −log_10_ (*P* values). A significance threshold of *P*_FDR_ < 0.05 was applied. ENT, ear, nose, and throat.

**Figure S13. Correlation of performance metrics between the training and testing sets.**
Scatter plots showing the correlations of the C-index (A), NRI (B), and IDI (C) for the PCAS-based model between the training and testing sets. Each point represents one disease. C-index, Harrell’s concordance index; NRI, net reclassification improvement; IDI, integrated discrimination improvement.

**References**

Cao Z, Zheng X, Yang H et al. Association of obesity status and metabolic syndrome with site-specific cancers: a population-based cohort study. *Br J Cancer* 2020;**123**:1336-1344.

Chudasama YV, Khunti K, Gillies CL et al. Healthy lifestyle and life expectancy in people with multimorbidity in the UK Biobank: A longitudinal cohort study. *PLoS Med* 2020;**17**:e1003332.

Deng YT, You J, He Y et al. Atlas of the plasma proteome in health and disease in 53,026 adults. *Cell* 2025;**188**:253-271.e7.

Fix E,Hodges JL. Discriminatory Analysis. Nonparametric Discrimination: Consistency Properties. *International Statistical Review / Revue Internationale de Statistique* 1989;**57**:238-247.

Folkersen L, Fauman E, Sabater-Lleal M et al. Mapping of 79 loci for 83 plasma protein biomarkers in cardiovascular disease. *PLoS Genet* 2017;**13**:e1006706.

Glaesmer H, Schulz A, Häuser W et al. The childhood trauma screener (CTS) - development and validation of cut-off-scores for classificatory diagnostics. *Psychiatr Prax* 2013;**40**:220-6.

Grabe HJ, Schulz A, Schmidt CO et al. A brief instrument for the assessment of childhood abuse and neglect: the childhood trauma screener (CTS). *Psychiatr Prax* 2012;**39**:109-15.

Soares ALG, Hammerton G, Howe LD et al. Sex differences in the association between childhood maltreatment and cardiovascular disease in the UK Biobank. *Heart* 2020;**106**:1310-1316.

Sudlow C, Gallacher J, Allen N et al. UK biobank: an open access resource for identifying the causes of a wide range of complex diseases of middle and old age. *PLoS Med* 2015;**12**:e1001779.

Suhre K, Arnold M, Bhagwat AM et al. Connecting genetic risk to disease end points through the human blood plasma proteome. *Nat Commun* 2017;**8**:14357.

Sun BB, Chiou J, Traylor M et al. Plasma proteomic associations with genetics and health in the UK Biobank. *Nature* 2023;**622**:329-338.

Sun BB, Maranville JC, Peters JE et al. Genomic atlas of the human plasma proteome. *Nature* 2018;**558**:73-79.

Warrier V, Kwong ASF, Luo M et al. Gene-environment correlations and causal effects of childhood maltreatment on physical and mental health: a genetically informed approach. *Lancet Psychiatry* 2021;**8**:373-386.

Wik L, Nordberg N, Broberg J et al. Proximity Extension Assay in Combination with Next-Generation Sequencing for High-throughput Proteome-wide Analysis. *Mol Cell Proteomics* 2021;**20**:100168.

Zhang YB, Chen C, Pan XF et al. Associations of healthy lifestyle and socioeconomic status with mortality and incident cardiovascular disease: two prospective cohort studies. *Bmj* 2021;**373**:n604.
